# GLP1R and GIPR expression and signaling in pancreatic alpha cells, beta cells and delta cells

**DOI:** 10.1016/j.peptides.2024.171179

**Published:** 2024-02-14

**Authors:** Ali H. Shilleh, Katrina Viloria, Johannes Broichhagen, Jonathan E. Campbell, David J. Hodson

**Affiliations:** 1 Oxford Centre for Diabetes, Endocrinology and Metabolism (OCDEM), NIHR Oxford Biomedical Research Centre, Churchill Hospital, Radcliffe Department of Medicine, University of Oxford, Oxford, UK; 2 Leibniz-Forschungsinstitut für Molekulare Pharmakologie, Berlin, Germany; 3 Duke Molecular Physiology Institute, USA; 4 Department of Medicine, Division of Endocrinology, Duke University, Durham, NC, USA; 5 Department of Pharmacology and Cancer Biology, Duke University, Durham, NC, USA

**Keywords:** GLP1R, GIPR, insulin, glucagon, somatostatin, islet, alpha cell, beta cell, delta cell

## Abstract

Glucagon-like peptide 1 receptor (GLP1R) and glucose-dependent insulinotropic polypeptide receptor (GIPR) are transmembrane receptors involved in insulin, glucagon and somatostatin secretion from the pancreatic islet. Therapeutic targeting of GLP1R and GIPR restores blood glucose levels in part by influencing beta cell, alpha cell and delta cell function. Despite the importance of the incretin-mimetics for diabetes therapy, our understanding of GLP1R and GIPR expression patterns and signaling within the islet remain incomplete. Here, we present the evidence for GLP1R and GIPR expression in the major islet cell types, before addressing signaling pathway(s) engaged, as well as their influence on cell survival and function. While GLP1R is largely a beta cell-specific marker within the islet, GIPR is expressed in alpha cells, beta cells, and (possibly) delta cells. GLP1R and GIPR engage G_s_-coupled pathways in most settings, although the exact outcome on hormone release depends on paracrine communication and promiscuous signalling. Biased agonism away from beta-arrestin is an emerging concept for improving therapeutic efficacy, and is also relevant for GLP1R/GIPR dual agonism. Lastly, dual agonists exert multiple effects on islet function through GIPR > GLP1R imbalance, increased GLP1R surface expression and cAMP signaling, as well as beneficial alpha cell-beta cell-delta cell crosstalk.

## 1 Introduction

Glucagon-like peptide 1 receptor (GLP1R) and glucose-dependent insulinotropic polypeptide receptor (GIPR) are class B G protein-coupled receptors (GPCRs) belonging to the secretin family [1]. Following oral ingestion of food, glucagon-like peptide 1 (GLP1) and glucose-dependent insulinotropic polypeptide (GIP) are released from intestinal L- and K-cell [2, 3], respectively, before engaging their cognate receptors. The large amplification of insulin secretion observed in response to oral glucose but not intravenous glucose- i.e. the “incretin effect”- is largely dependent upon GLP1R and GIPR activation in the pancreatic islets [4, 5]. For an in-depth historic narrative of the discovery of the incretin concept, and development of the incretin-mimetic drug class, we refer the reader to authoritative reviews elsewhere [6–9].

Despite the impressive volume of work on GLP1R and GIPR signaling in the context of therapeutic targeting (reviewed in [10–12]), we are still only just beginning to grasp how GLP1 and GIP might regulate alpha cell, beta cell and delta cell function to maintain glucose homeostasis. Moreover, lack of specific, validated antibodies has up until now precluded our understanding of GIPR and GLP1R expression, meaning that the cell targets for GLP1RA and GIPRA have been difficult to assign. Lastly there are a number of open questions in the field, for example are GLP1R/GIPR plastic across physiological state/lifespan, what is the role in intraislet paracrine signaling in determining the incretin effect, and do major specific differences exist with respect to GLP1R/GIPR signaling?

The aim of the current article is thus to provide a high-level view of GLP1R and GIPR expression and signalling in the pancreatic islet, with relevance to hormone secretion, cell survival and glucose homeostasis. While much work has been performed in heterologous cell systems expressing GLP1R and GIPR, we will use examples pertinent to primary tissues wherever possible to increase relevance for islet biology.

## 2 GLP1R expression patterns

Table 1 summarizes GLP1R expression patterns and levels in the islet.

Single-cell screening studies, as well as studies on purified alpha cell, beta cell and delta cell fractions, have provided the first insight into *Glp1r*/*GLP1R* expression levels across the islet. Thus, *GLP1R*/*Glp1r* transcripts are highly expressed in beta cells, with some expression in delta cells depending upon dataset, but always low to absent expression in alpha cells [13–18]. However, GPCRs as low abundance transcripts suffer from apparent heterogeneity and drop out in earlier single-cell screening studies [19], meaning that lack of detection does not equal lack of expression. Nonetheless, analyses of primary islets/cells from Glp1rCre reporter mice expressing fluorescent lineage labels confirm the accuracy of single cell-screening studies for *GLP1R*/*Glp1r*. In these mice, any cell that has switched-on Cre will be indelibly labelled for the lifetime of that cell, subject to Cre expression faithfully recapitulating *Glp1r* expression. By using a CAG promoter to drive reporter expression from the Rosa26 locus, this model allows high fidelity identification of cells that express even very low levels of *Glp1r*. Using this model, two independent studies showed that Glp1r expression is confined to beta cells and delta cells, and is undetectable in most alpha cells (~ 5% expression) [20, 21]. It should be noted however that reporter approaches might label cells that were *Glp1r+*, but which have now adopted a new cell fate, and only report transcript and not protein expression.

Confirming GLP1R and GIPR expression at the protein level has remained more challenging. GPCRs as 7-transmembrane proteins are expressed at relatively low levels, and require detergent to stabilize [22], which can mask extracellular epitopes needed for antibody generation. Despite numerous studies showing GLP1R immunostaining in the pancreas, many studies are now known to have used antibodies that bind non-specifically to non-GLP1R epitopes. Through careful and rigorous testing, four antibodies are now confirmed to specifically target human/primate/rat/mouse/dog GLP1R, as tested using gold-standard validation such as *Glp1r*^-/-^ tissue, or cells transfected with GLP1R vs. mock plasmid [23–28] (reviewed in [29]). Using validated antibodies, a number of studies reported beta cell- and delta cell-specific GLP1R expression in mouse and tissue [26, 30], confirming single cell screening data.

To directly link gene expression levels with protein expression, Gray et al labelled dissociated islet cells from Glp1rCre reporter mice with monoclonal antagonistic antibody, before sorting cells and quantifying promoter activity, mRNA expression and protein expression [15]. Using this approach, transcript and protein expression was abundant in beta cells, but undetectable in alpha cells. Unexpectedly, while transcript was present in delta cells, no protein could be detected. Thus, these studies suggest caution when extrapolating single cell screening data to the protein level, and suggest that post-translational regulation/stability of GLP1R expression in delta cells might differ from beta cells. Alternatively, there could be a sensitivity issue due to the relative lack of delta cells for analysis, or isolation of delta cells from the islet context with e.g. concomitant decreases in GLP1R expression levels. *GLP1R/Glp1r* mRNA might also exert other regulatory functions in delta cells, which prevents translation initiation.

Single cell screening, reporter approaches, and antibody staining are unable to report how therapeutically-relevant ligands access and bind GLP1R/GIPR. To allow this, we developed and validated antagonistic chemical probes, LUXendins, that fluorescently label and report orthosteric GLP1R binding [31, 32]. The probes, which comprise of the high affinity/potency antagonist Exendin4(9-39) modified at the C-terminus to allow bioconjugation with fluorophore, are highly specific as shown using CRISPR-deleted *Glp1r*^-/-^ mice that encode no detectable protein [31, 32]. Using LUXendins, we quantified cell type-specific labelling in isolated islets, and showed that beta cells express abundant protein, with ~20% non-beta cells expressing GLP1R attributed to either occasional alpha cells (and in keeping with reporter approaches) or delta cells [31]. A caveat here is that assigning cell membrane staining in closely opposed cell populations is notoriously difficult, which could lead to false assignment of GLP1R+ cells, especially those completely surrounded by beta cells (i.e. delta cells). Studies on FAC-sorted cells are ongoing.

Lastly, it should be noted that GPCRs are at the head of a highly-amplified signaling cascade, and lack of detection is not evidence of absence. More sensitive approaches, for example fusing tags (e.g. SNAP and Halo-tag), enzymes (e.g. BioID, APEX) or well-validated epitopes (e.g. HA- or ALFA-tag) [33, 34] to GLP1R (and GIPR), are warranted in the future to improve the detection limit, and categorically rule out or in expression. Furthermore, ligand-receptor binding studies in sorted cell fractions are needed to demonstrate that differing expression levels of GLP1R from low to high are equally signaling-competent.

## 3 Plasticity of GLP1R expression

GLP1R expression plasticity is not well studied. In multiparous mice, *Glp1r* mRNA was found to be down-regulated, although GLP1R protein expression was no different to nulliparous control mice [15]. In the same studies, mice fed high fat diet for 4 weeks showed no changes in *Glp1r* mRNA or GLP1R protein expression [15]. While 4-week high fat diet is sufficient to induce beta cell proliferation [35, 36], more aggressive or longer insults might be needed to influence GLP1R mRNA and protein expression. Indeed, studies in 90% pancreatectomized rats, which display profound hyperglycemia, showed downregulation of *Glp1r* mRNA expression, confirmed also using a 96-hour hyperglycemic clamp. Similarly, exposure of MIN6 cells to high glucose (25 mM) leads to decreases in *Glp1r* mRNA and cell surface expression [37]. While incubation of INS1E and MIN6 cells with the fatty acid palmitate reduced *Glp1r* mRNA expression [38], the opposite is seen in human islets with increased *Glp1r* mRNA and GLP1R protein expression [39]. However, human islet GLP1-responses are negatively correlated with donor BMI [39], and lipid infusion selectively impairs the incretin effect in healthy human participants [40], suggesting that any increases in *Glp1r*/GLP1R are uncoupled from downstream signaling pathways in vitro and in vivo.

## 4 GIPR expression patterns

Table 2 summarizes GIP1R expression patterns and levels in the islet.

Based upon single cell screening, or purified cell analysis, *GIPR* transcripts are expressed across alpha cells, beta cells and delta cells, with highest expression in alpha cells and beta cells [13–18]. There appears to be good consensus between mouse and human datasets [13–18]. To the best of our knowledge, no specific, validated antibodies exist in the academic setting for GIPR, making detection even more challenging than for GLP1R. To circumvent this issue, we recently developed stabilized agonist chemical probes, sGIPs, that fluorescently label and report GIPR across different tissues including islets and brain [41]. Demonstrating specificity, studies in GIPR^-/-^ mice showed absence of cFOS activation in the hypothalamus following iv injection with sGIP. Furthermore, sGIP failed to label beta cells specifically deleted for GIPR (*MIP-Cre;GIPR*^*fl/fl*^) [41]. Using LUXendin to counter-label beta cells (and possibly delta cells) via GLP1R, we were able to establish strong GIPR protein expression in both the alpha cell and beta cell compartments [41], although we were unable to look at GIPR in delta cells in the same study.

## 5 Plasticity of GIPR expression

There is no reliable data on GIPR protein expression plasticity due to lack of specific antibodies. However, at the mRNA level, *Gipr* expression was unchanged in INS1E and MIN6 cells following 24 hr treatment with palmitate [38]. This finding might however reflect the glucose levels used, since palmitic acid + 5.5 mM glucose doubled *Gipr* mRNA levels in INS1E cells [42]. In the same studies, exposure of INS1E cells to glucotoxicity was found to decrease Gipr expression [42]. In pancreatectomized rats, *Gipr* mRNA expression was lowered, whereas there was an increase in response to prolonged hyperglycemic clamp [43]. Finally, *Gipr* mRNA was found to be reduced by 60% in islets from *db/db* mice, corresponding to an inability of exogenous GIP to lower glycemia during a glucose tolerance test [44].

## 6 GLP1R/GIPR expression in human islets

Most of the available data on GLP1R and GIPR expression patterns are from experiments in rat or mouse islets. Studies in primate and human islets using fluorescent staining showed strong co-localization with insulin, although alpha cells/delta cells were unlabelled, and statistical quantification was not performed [23]. An earlier study using a discontinued (but recently continued) antibody showed GLP1R expression restricted solely to INS+ and not SST+ or GCG+ cells [26]. While the antibody in question was validated in mouse GLP1R^-/-^ tissue, it is worth noting that antibody staining was mainly cytoplasmic, while the GLP1R is largely present at the cell membrane in its non-stimulated state (as shown using other validated antibodies). More recent studies used non-fluorescent immunostaining to investigate GLP1R expression patterns in resected pancreata from individuals with insulinoma, pancreatitis or pancreatic adenocarcinoma [45]. Strong staining was seen in both the INS+ and SST+ compartments, although serial sections needed to be used, limiting three-dimensional visualization. Lastly, confocal imaging in normal human pancreas showed predominant immunostaining in beta cells, with some labelling of delta cells [46]. In summary, GLP1R appears to be primarily in beta cells in human islets, with expression in occasional delta cells, warranting further quantitative studies.

## 7 GLP1R signaling and function in beta cells

**Figure 1:**
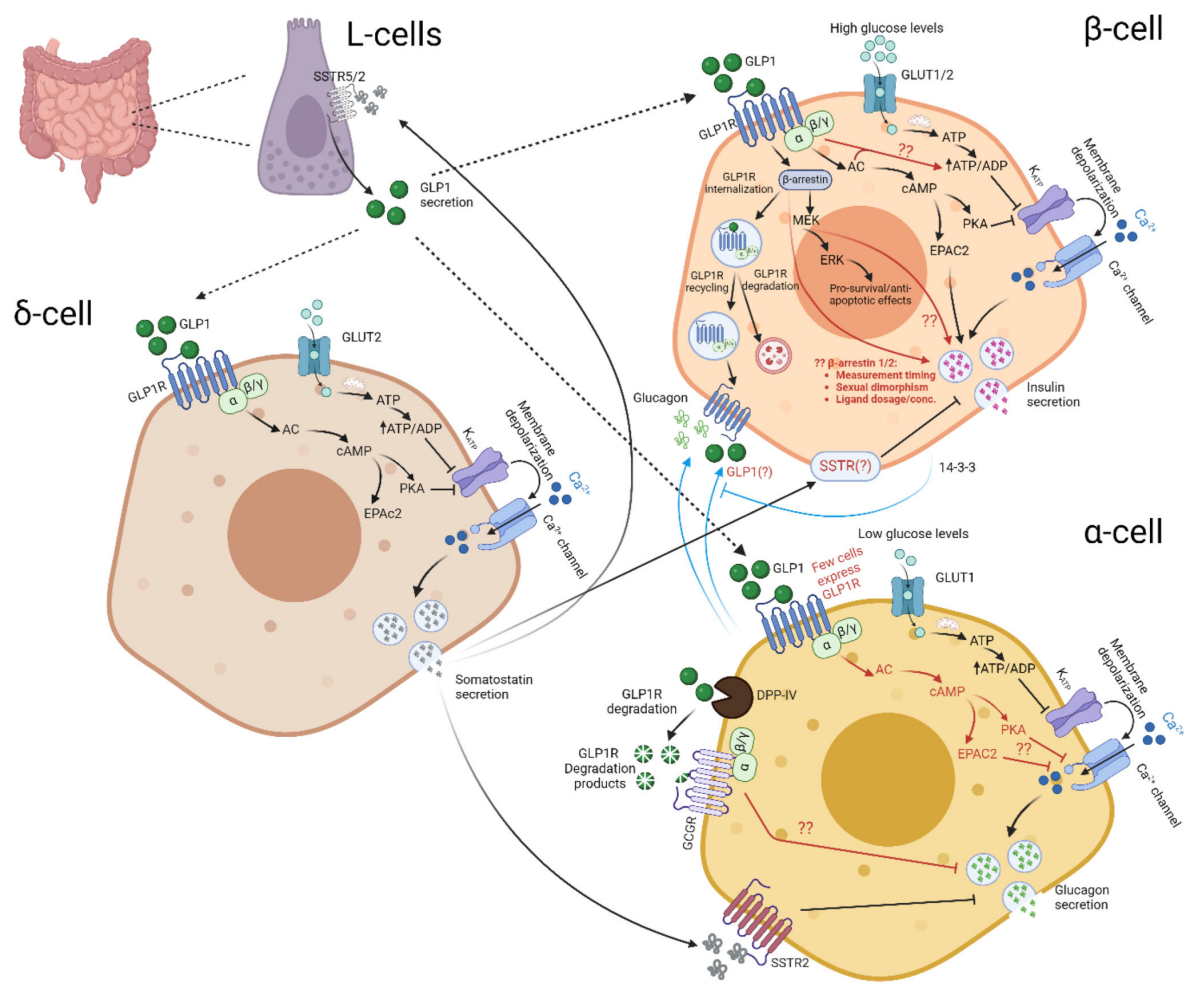
Schematic of GLP1R signalling pathways in pancreatic beta cells, alpha cells and delta cells. Black arrows highlight well-studied pathways downstream of GLP1R activation, while red arrows highlight pathways that have yet to be fully established. Blue arrows indicate paracrine inputs that are influenced by, or influence GLP1R signaling.

The major beta cell GLP1R signaling pathways are highlighted in Figure 1.

Single cell-screening, reporter allele, antibody and fluorescent probe studies have all established that GLP1R is abundantly expressed in beta cells, with some possible expression in delta cells. Such localization pattern fits with the effects of GLP1R agonism to potently increase glucose-dependent insulin release. Following GLP1RA binding, GLP1R rapidly (milliseconds) arrests at the cell membrane [47], before engaging Gαs to activate adenylate cyclase and cAMP generation [48, 49]. By interacting with PKA and Epac, cAMP increases Ca^2+^ influx through voltage-dependent Ca^2+^ channels, and increases the competency of insulin vesicles to undergo Ca^2+^-dependent exocytosis [50–52]. While this is the major pathway through which GLP1R potentiates insulin secretion, there might be more minor contributions from other pathways such as MEK-ERK [53, 54], as well as via changes in cytosolic ATP/ADP ratio and beta cell metabolism [55, 56]. By contrast, MEK-ERK is likely to be more important for the beta cell pro-survival/anti-apoptotic effects of GLP1RA [57], alongside cAMP-PKA [58].

Post-stimulation, GRK phosphorylates serine and threonine residues on the GLP1R C-terminal tail, which leads to recruitment of beta-arrestin(s), desensitization and endocytosis (internalization) (reviewed in [59]). In the endosomal compartment, GLP1R will then: 1) continue to signal via sustained endosomal cAMP generation; 2) traffic back to the membrane as ‘recycled’ GLP1R; or 3) be degraded via lysosomes. GLP1R trafficking is therapeutically-relevant, since ‘biased’ agonists that reduce beta-arrestin recruitment appear to be more effective at stimulating sustained insulin secretion [60]. The exact role of beta-arrestin in GLP1R signaling remains poorly defined. Beta cell-specific deletion of beta-arrestin 1 was found to reduce Epac2 engagement by sulfonylureas, limiting their effects on insulin release, but was without effect on GLP1/GLP1RA-stimulated insulin release [61]. Similarly, while beta cell-specific deletion of beta-arrestin 2 impaired glucose-stimulated insulin secretion and decreased beta cells mass, there was no effect on GLP1/GLP1RA-stimulated insulin secretion [62, 63]. More recent studies have shown that beta-arrestin 1 might in fact restrain GLP1R signaling, since its deletion from the beta cell compartment amplifies insulin secretory responses to GLP1/GLP1RA [64]. Similarly, beta arrestin 2 knockout has been shown to impair cAMP responses to GLP1RA, a defect that can be rescued by beta arrestin 1 silencing [65].

Suggesting that some of the discrepancy between studies on beta arrestin might be due to measurement timings and sexual dimorphism, beta-arrestin 2 knockout slightly improved glucose tolerance in male animals 6 hours post-injection of Ex4, whereas glucose tolerance was markedly impaired in female animals 30-60 mins post-injection [66]. Ligand and concentration might also explain some of the conflicting findings, since at lower picomolar concentrations of GLP1, loss of beta-arrestin 1 from the beta cell compartment enhances insulin secretion [64]. Likewise, at 10-100 pM GLP1, beta-arrestin 2 uncouples cAMP from PKA, decreasing insulin secretion [67]. By contrast, at 1 nM GLP1 beta-arrestin 2 is required for GLP1R to signal via ERK/CREB [67]. Thus, the consensus view is that beta-arrestin 2 is important for GLP1RA-stimulated insulin secretion, but that this effect is highly context-dependent (concentration, ligand used, sex, timing). Beta-arrestin is therapeutically relevant, since GLP1RA modified to decrease beta-arrestin recruitment (“biased agonists”) display greater GLP1R surface retention, increased insulin secretion over the long-term, as well as faster ligand dissociation rates [68]. Furthermore, substantive rather than moderate reduction in beta-arrestin recruitment is required for the superior efficacy of biased agonists versus semaglutide [69]. Again, pointing to an important role of GLP1 levels in GLP1R signaling, picomolar concentrations of the ligand have been shown to recruit Gαq instead of Gαs, activating PLC-PKC instead of cAMP-PKA-ERK [70]. Similarly, chronic beta cell depolarization via either elevated glucose or K_ATP_-channel inactivating mutations, leads to a switch from Gαs to Gαq signaling [71].

## 8 The tissue context and beta cell GLP1R signaling

Most studies have necessarily focused on signaling in single beta cells. It should be noted however that the tissue context endows GLP1R signaling with a tertiary level of regulation. Thus, in the human islet, GLP1 engages an electrotonically-coupled beta cell subnetwork, which provides highly coordinated cell activation [39]. By contrast, in healthy rodent islets, GLP1 increases Ca^2+^ duty cycle (i.e. ON time), although during high fat diet a beta cell subnetwork, analogous to that in human islets, becomes more predominant [39]. Most recently, we have shown that not all beta cells are recruited equally into GLP1R trafficking, with three subpopulations, defined by rapid, moderate and slow recruitment, differentially engaged by the different agonist classes [47]. Exendin4 equally stimulates all three beta cell subpopulations, whereas semaglutide and tirzepatide tend to rely more on the moderate and slow subpopulations [47]. The reasons for this remain unknown, but we speculate that engagement of beta cell subpopulations might increase long term treatment efficacy, by allowing cells to recover from prolonged GLP1R stimulation.

## 9 Alpha cell proglucagon products and beta cell GLP1R signaling

Although active GLP1 is predominantly derived from the gut, alpha cells can also produce and locally secrete GLP1 via expression PC1/3 [72–74]. Studies have shown that GLP1 can be detected in islet supernatant or protein extracts [73, 75], and GLP1 expression and release increase in both rodent and human islets during metabolic stress and T2D [72, 76], which suggest the existence of a compensatory pathway to maintain beta cell function. Supporting a (bidirectional) role of alpha cell-derived proglucagon products in beta cell GLP1R signaling are the observations that glucose-stimulated insulin secretion is reduced by GLP1R antagonism or beta cell specific GLP1R deletion [72, 77–79], and beta cell GLP1R activation induces GLP1 expression in a subset of alpha cells that adopt beta cell-like features [73, 80]. Assigning GLP1 as a major driver of alpha cell -> beta cell GLP1R signaling is, however, complicated by glucagon signaling promiscuously through GLP1R [77–79]. Given the relative abundance of glucagon versus alpha cell-derived GLP1, it can be argued that glucagon action at the GLP1R is likely to outweigh that of GLP1. More work is needed to dissociate and quantify effects of GLP1 versus glucagon on beta cell GLP1R signaling.

## 10 GLP1R signaling in alpha cells

The major alpha cell GLP1R signaling pathways are highlighted in Figure 1.

Treatment with GLP1RA dramatically reduces glucagon release, despite the low expression levels of GLP1R in alpha cells. Patch clamp electrophysiology studies have shown that GLP1 inhibits N/L and P/Q-type voltage-dependent Ca^2+^ channels (VDCC) in mouse [81] and human [82] alpha cells, respectively, to lower glucagon release. Invoking an important role for cAMP in alpha cell GLP1R signaling, the glucagonostatic effect of GLP1 can largely be prevented by PKA inhibition [81, 82], although there is a “PKA inhibition resistant” fraction, which depends upon Epac2 signaling [81]. Suggesting that GLP1 directly influences alpha cell activity, rather than acts via beta cells or delta cells, no suppression of glucagon release was detected in the presence of insulin receptor or somatostatin receptor 2 blockade [82]. These findings imply that GLP1R in alpha cells might be under the detection limit for even the most sensitive visualization strategies (e.g. (i.e. Cre-dependent reporter labelling)), yet still capable of signaling.

Another, more plausible explanation is that GLP1 degradation products are able to signal via the glucagon receptor. GLP1 is rapidly degraded by DPP-IV, which is present on the surface of most islet cell types (i.e. alpha, beta, delta). While both GLP1 7-36 and GLP1 9-36 inhibit glucagon secretion at pM concentration, pre-incubation with DPP-IV abolished responses to GLP1 7-36 but not GLP1 9-36 [83]. These data suggest that GLP1 7-36 is the propeptide for GLP1 9-36, and that GLP1 9-36 is the major signaling ligand. Notably, the glucagonostatic effect of GLP1 9-36 is still present in *Glp1r*^-/-^ knockout islets, but is absent from *Gcgr*-/- knockout islets [83]. Together, these results show that GLP1 degradation products promiscuously activate the GCGR in alpha cells to suppress glucagon secretion, and might provide a valuable target for reducing hyperglucagonemia. While alpha cells express very low levels of GCGR, it is still thought to be adequate for signalling due to the relative strength of Gi coupling [83]. However, it still remains unknown how GLP1RA exert glucagonostatic effects, since their resistance to degradation makes promiscuous signaling via the GCGR unlikely. Furthermore, studies are required to uncover the paracrine beta cell (or possibly delta cell)→alpha cell circuits that might underlie this divergence between the actions of endogenous GLP1 and GLP1RA on glucagon secretion.

## 11 GLP1R signaling and function in delta cells

The major delta cell GLP1R signaling pathways are highlighted in Figure 1.

GLP1 has been shown to stimulate somatostatin release in isolated islets as well as the perfused pancreas [84, 85]. While the exact signaling pathways underlying this effect have not been established, cAMP-raising agents such as forskolin similarly elevate somatostatin secretion, and application of PKA and Epac2 inhibitors reduces glucose-stimulated somatostatin secretion [86]. Thus, cAMP pathways are active in delta cells, contribute to their glucose-regulation, and as such might also be engaged by GLP1R. Of note, GLP1-stimulated somatostatin release inhibits glucagon secretion in a paracrine manner, prevented by antagonizing SSTR2 [46, 84, 85], which is expressed largely on alpha cells in the mouse islet [13, 14, 87], but on both alpha cells and beta cells in the human islet [88]. In addition, GLP1RA counteracts the glucagon-raising effects of SGLT2 inhibitors via somatostatin release and SSTR2 signaling [46]. Thus, delta cell-regulation of alpha cells could constitute a potentially important route for the glucagonostatic effects of GLP1RA. Recent studies have also shown that release of somatostatin, presumably from delta cells, predominantly activates SSTR5a > SSTR2 to increase GLP1 release from the gut, which then feeds back at the level of the pancreas to improve glycemia in a GLP1R-dependent manner [89]. Since somatostatin is also a known negative regulator of insulin secretion [90], GLP1R likely stimulates delta cell->beta cell, as well as delta cell->alpha crosstalk. While it is clear that GLP1RA influence delta cell activity and somatostatin release, it is less clear whether this is direct or indirect. Given that the consensus for GLP1R expression on delta cells has not been reached, both mechanisms should be considered. To this end, conditional deletion of the GLP1R in delta cells might be helpful.

## 12 GIPR signaling in beta cells

**Figure 2:**
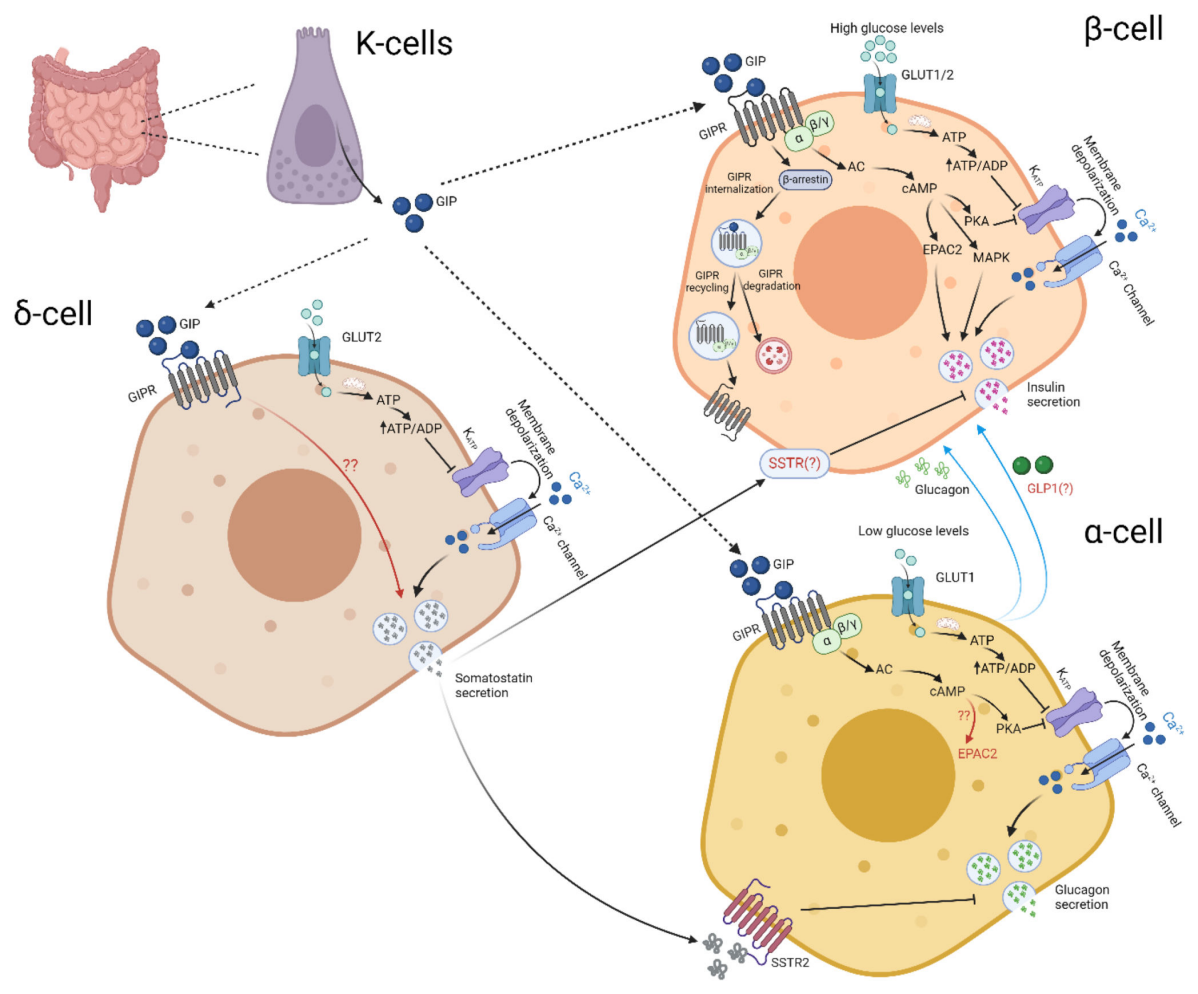
Schematic of GIPR signalling pathways in pancreatic beta cells, alpha cells and delta cells. Black arrows highlight well-studied pathways downstream of GIPR activation, while red arrows highlight pathways that have yet to be fully established. Blue arrows indicate paracrine inputs that are influenced by GIPR signaling, or influence GLP1R signaling.

The major beta cell GIPR signaling pathways are highlighted in Figure 2.

GIPR signals similarly to GLP1R, with ligand-induced Gαs recruitment leading to adenylate cyclase activation, PKA/cAMP/Epac activation, and Ca^2+^-dependent amplification of insulin release (with contributions from MAPK [91]). While abundant data exists on GLP1R signaling in MIN6/INS1 cells, as well as primary beta cells, it should be noted that most data on GIPR signaling is extrapolated from heterologous cell systems and might not necessarily reflect the situation in the beta cell itself. While GLP1R is able to internalize in the absence of beta-arrestin 2, GIPR endocytosis requires expression of the scaffold protein [67, 68]. In addition, major differences exist in spatiotemporal signalling between GLP1R and GIPR in primary islets, with the latter ligand displaying reduced ligand-induced desensitization and internalization, as well as membrane recycling [65].

In healthy adults, GIP disproportionately stimulates insulin secretion versus GLP1, studied using antagonists [92] (with some caveats [93]). However, this observation might reflect ligand availability or relative receptor expression, since in isolated islets GLP1 and GIP stimulate insulin secretion to a similar extent, taking into account their respective potencies [77]. While studies in humans show additive effects of GIP and GLP1 on insulin secretion during health [94], but not in T2D [95], this has yet to be fully tested in a system where beta cell activity can be interrogated in isolation. As for GLP1, GIP-stimulated insulin secretion is glucose-dependent, with increased insulin stimulation at higher glucose levels. Glucose, however, is not the only permissive signal, with postprandial amino acid flux also contributing. Indeed, the insulinotropic effect of GIP partly depends upon paracrine interactions between alpha cells and beta cells, since the action of alanine to potentiate GIP-stimulated insulin secretion is lost in alpha cell *Gipr*^-/-^ islets [96].

## 13 GIPR signaling in alpha cells

The major alpha cell GIPR signaling pathways are highlighted in Figure 2.

Alpha cells abundantly express GIPR, suggesting a direct mode of GIP action unlike for GLP1R. Studies in the alphaTC1 alpha-cell line as well as isolated alpha cells have shown that GIP agonism increases cytosolic cAMP levels [97, 98]. In rat alpha cells, GIP increases Ca^2+^ currents and potentiates glucagon secretion stimulated by voltage-clamp depolarization, an effect prevented the PKA inhibitor Rp-8-Br-cAMPS [99]. Studies in a number of models, from cell lines to isolated islets to perfused pancreas [96, 98, 100–102], have shown that GIP potently stimulates glucagon secretion in alpha cells at low (< 5 mM) glucose levels, which can be prevented with a GIPR antagonist. In healthy individuals and those with type 1 diabetes, GIP infusion stimulates glucagon but not insulin secretion during hypoglycemia and euglycemia [103–105]. Thus, as for its effects on insulin secretion, GIP stimulates glucagon secretion in a glucose-dependent manner. Recent studies however suggest that the effect of GIP-alone on glucagon secretion at low glucose is relatively small, and that postprandial increases in amino acids such as alanine are required for the full potentiating effect of GIP [96]. Of note, the same studies showed that alanine also potentiates GIP-stimulated glucagon secretion at high glucose, although this is ~10-fold less than at low glucose. These effects of GIP are dependent on GIPR signalling, since they are absent in *Gipr*^-/-^ alpha cells [96]. Lastly, GIP-induced glucagon secretion augments insulin secretion, an effect further amplified by prior application of alanine, but abolished by alpha cell-specific GIPR deletion i.e. GIP engages positive alpha cell->beta cell crosstalk via the alpha cell GIPR [96].

## 14 GIPR signaling in delta cells

The major delta cell GIPR signaling pathways are highlighted in Figure 2.

There is a paucity of data on GIPR signaling and delta cell function. Seminal studies almost 40 years ago showed that GIP is able to stimulate somatostatin at 1 nM, with increased efficacy at 10 nM [106]. Since both 1 nM and 10 nM equipotently stimulate insulin secretion [106], it is likely that either GIPR expression, GIPR affinity for ligand, or intracellular pathway coupling is lower in delta cells than in beta cells. In the perfused rat pancreas, application of 10 nM GIP leads to an ~7-fold increase in somastostatin secretion, which is similar in magnitude to that evoked by GLP1 [85]. As for GLP1R, the GIPR signaling pathways involved remain unknown but likely involve cAMP given its known role in glucose-suppression of somatostatin secretion [90]. As for GLP1R, GIPR activation is likely to engage both delta cell->beta cell and delta cell->alpha crosstalk, given the known roles of somatostatin in negative regulation of insulin and glucagon release [90].

## 15 Dual GLP1R and GIPR agonism in the islet

**Figure 3:**
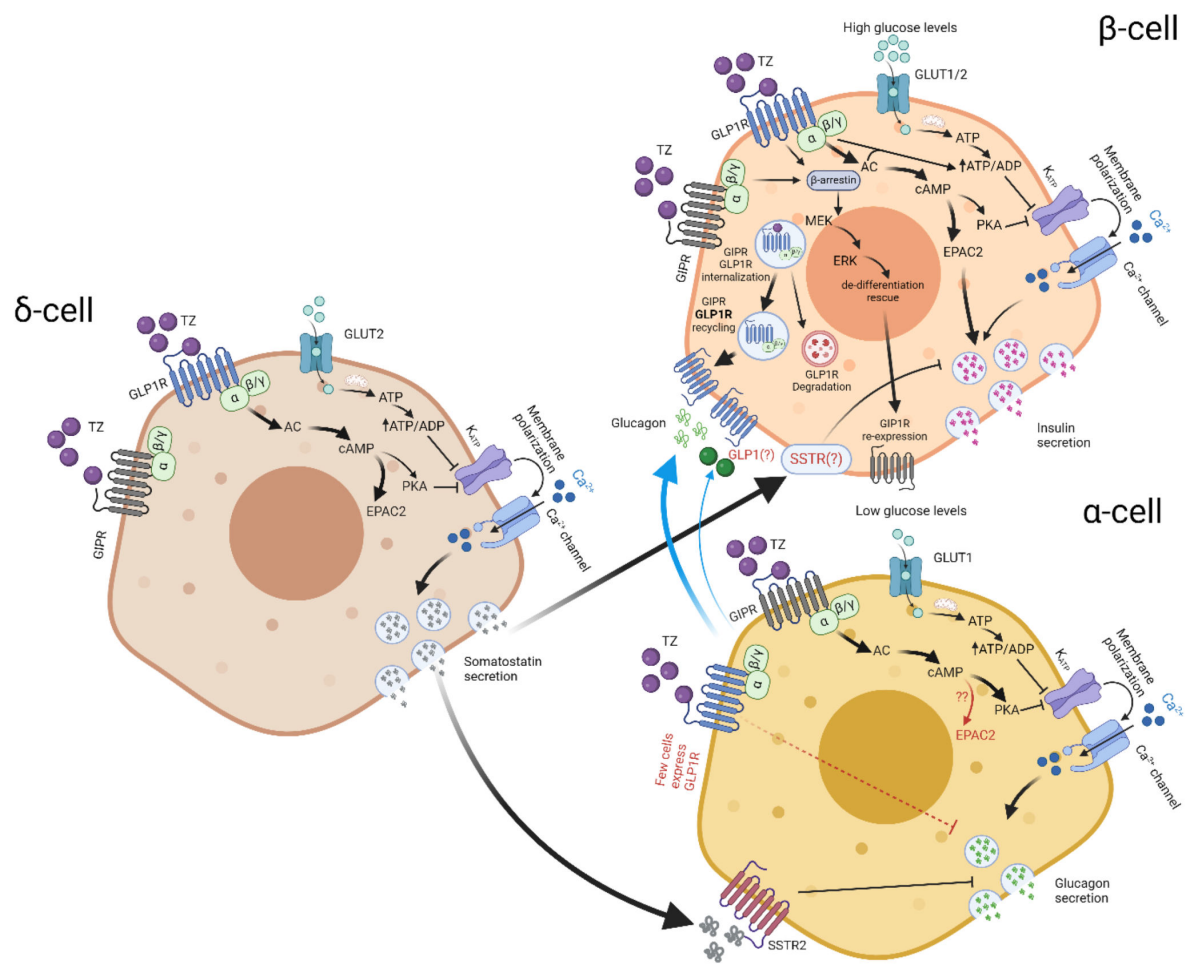
Schematic of tirzepatide signalling pathways in pancreatic beta cells, alpha cells and delta cells. Black arrows highlight well-studied pathways downstream of GLP1R and GIPR activation, while red arrows highlight pathways that have yet to be fully established. Blue arrows indicate paracrine inputs that are influenced by GLP1R/GIPR signaling, or influence GLP1R signaling.

Figure 3. highlights the major tirzepatide (dual GLP1R/GIPR agonist) signaling pathways within the islet.

GLP1R and GIPR signalling are generally viewed in isolation. However, GIP and GLP1 are both simultaneously elevated in healthy adults during the post-prandial phase, have similar degradation kinetics, and so it stands to reason that GLP1R and GIPR rarely signal in isolation. If GLP1R and GIPR co-activation occur during normal physiology, why was GLP1RA largely the focus of T2D treatment? While early studies demonstrated the clinical potential of GIPRA in rodent models of type 2 diabetes/obesity [107, 108], development of GIP agonists for human use was largely curtailed by GIPR downregulation, the lack of glycemic benefit of GIPRA, as well as the concept that GIPR agonism drives obesity [109]. However, it was recognized that a number of actions of GIPRA might potentially synergize with GLP1RA. For example, the glucagonostatic effects of GLP1RA might counteract the glucagontropic effects of GIPRA, whereas the anti-emetic effects of GIPRA might counteract the pro-emetic effects of GLP1RA. Thus, unimolecular multireceptor agonists were generated containing components of GLP1 and GIP (NNC0090-2746), with unnatural amino acids and C-terminal extension for stability, or alternatively a GIP sequence modified to have GLP1R binding affinity (tirzepatide) [110, 111]. While NNC0090-2746 was superior to liraglutide, it did not outperform once-weekly semaglutide and as such trials were discontinued [12, 112]. By contrast, tirzepatide was non-inferior and superior to the GLP1RA for glycated hemoglobin [113]. It is now known that the imbalance of tirzepatide for GIPR >> GLP1R, as well as the mid-position of the fatty diacid moiety, probably lend the molecule to superior effects on glycemia (and obesity, which is covered elsewhere) [64, 114].

In terms of receptor signaling, tirzepatide is a full agonist at human (but not mouse) GIPR, but biases GLP1R away from beta arrestin, leading to increased GLP1R surface retention and cAMP signaling [64, 115]. In mouse islets, the effects of tirzepatide are largely mediated by GLP1R, whereas in human islets GIPR antagonism reduces tirzepatide-stimulated insulin secretion [114]. In addition, tirzepatide stimulates both glucagon and somatostatin secretion in human islets, in keeping with the known actions of both GIPRA and GLP1RA on delta cell function, but reflecting more the actions of GIPRA in alpha cells [114]. While the exact effects of dual agonists such as tirzepatide on the islet are poorly understood, it is likely that a number of features are at play to help normalize glycemia: 1) the GLP1R component rescues beta cell de-differentiation, allowing GIPR to be (re)-expressed; 2) GLP1R and GIPR agonism are now able to synergistically increase insulin secretion; 3) GIPR activation influences GLP1R beta-arrestin bias, increasing surface retention and improving cAMP generation; 4) imbalance toward GIPR allows some glucagon secretion, which would otherwise be nullified by the GLP1R component, increasing energy expenditure without major effects upon glycemia; 5) GIPR activates alpha cell→beta cell cross-talk to further increase insulin release; and 6) GIPR activates delta cell→beta cell and delta cell→alpha cell cross-talk, finely-regulating glucagon and insulin output.

While much work remains to understand how GLP1R-GIPR signaling influence islet function, dual agonists are already providing much needed insight into structure-activity relationships, signal bias and receptor cross-talk within the islet.

## 16 Future challenges

While GLP1R and GIPR signaling in beta cells is well characterized, relatively little is known in alpha cells and delta cells. For example, are GIPRA responses similar in alpha cells and beta cells, does this involve Gαs, PKA and cAMP signaling, and are beta arrestins involved in the endocytotic response? Likewise, do delta cells express GIPR/GLP1R in the intact islet? Do GIP1RA/GLP1RA engage canonical signaling pathways? What happens to islet hormone secretion when GIPR/GLP1R are specifically deleted in the delta cell compartment?

Given the known paracrine interplay between alpha cells, beta cells and delta cells, as well the increasingly apparent role of GLP1R and GIPR in mediating these interactions, it can be hypothesized that disruptions to any single cell type will disrupt at least two-three hormone axes [90, 96, 116, 117]. Furthermore, studies with dual agonists have shown that imbalance for GIPR > GLP1R is central to their efficacy versus GLP1RA-alone. How this imbalance might influence alpha cell and delta cell function, given that these cell types likely possess different surface expression profiles and levels of GLP1R and GIPR, remains unknown. Along similar lines, more work is needed to understand the relative contributions of alpha cell-derived GLP1 and glucagon to beta cell GLP1R signaling, and changes therein during metabolic stress. Mice harboring GLP1R mutated at key residues to recognise GLP1 but not glucagon might be valuable here.

Another challenge is detection of GLP1R and GIPR (and most GPCRs for that matter), which is required to fully assign cell targets. The current consensus view is that GLP1R is a beta cell-specific marker within the islet, with possible expression in delta cells but not alpha cells. GIPR is expressed both within beta cells and alpha cells, but delta cells remain to be investigated. There are various caveats with GLP1R/GIPR detection, including sensitivity of the approach used, protein versus transcript, vulnerability of some cell populations to dissociation/sorting, as well as the decline in expression levels during culture. It will be interesting to repeat studies in the future using highly amplified detection systems and/or genomic analysis to understand how cell-specific transcription factor networks impinge upon GLP1R and GIPR expression. Understanding ligand-receptor binding in the different cell populations also seems sensible given the contribution of bias and GIPR > GLP1R imbalance to (dual) agonist efficacy.

Most of our data on cell signaling is derived from heterologous cells systems where receptor is stably over-expressed. While representing a ‘clean’ and tractable experimental system, which is gold standard for pharmacological assessment, it is clear that cells within the islet do not exist in isolation. Thus, alpha cells release glucagon at high concentration, which binds GLP1R and GCGR on the beta cell surface to modulate insulin release [77, 78, 96, 118], beta cell-beta cell communication via gap junctions is critical for coordinated responses to stimulus (reviewed in [119]), and alpha cells and beta cells represent a heterogeneous transcriptomic and functional population [120, 121], with a high probability that this is also the case for delta cells. Studies in the intact tissue, under different stimulation regimes, as well as in response to different metabolic stressors, will be important for understanding how the tissue context influences GLP1R and GIPR signaling.

We expect these open questions to be addressed in the near future, since specific chemical probes exist for GIPR and GLP1R visualization, Cre-dependent cAMP reporter mice are available, GRKs and beta-arrestins can be inhibited/deleted, and GLP1R and GIPR can be conditionally knocked out. Over the next few years, we also expect GIPR antagonists, cell subtype specific ant(agonists) and quantitative omics, including proximity labelling, to be informative.

## 17 Summary

GLP1R and GIPR expression and signaling are clearly critical for alpha cell, beta cell and delta cell function, and as such regulation of islet hormone secretion and glucose homeostasis. Despite this, deletion of either GIPR or GLP1R results in only modest changes in oral glucose tolerance, probably due to upregulation of the other incretin axis [122]. However, in response to the same challenge, double GIPR and GLP1R knockout mice show much larger excursions in blood glucose, demonstrating the importance of both receptors for glycemic control [122]. Likewise, antagonism of GLP1R and GIPR leads to larger reductions in insulin secretion versus antagonism of either receptor alone [92]. The GLP1R and GIPR signalling mechanisms involved in these powerful homeostatic effects are well characterized in heterologous cell systems, but there are subtle differences in primary islets across species that we are only just starting to grasp. For example, intercellular communication within the islet determines hormone release and the glucose setpoint [116, 117], but also plays an important role in shaping responses to GLP1RA and GIPRA [96, 123]. The exact paracrine pathways involved are the focus of ongoing studies. Lastly, dual agonists exert complex effects upon GLP1R and GIPR signaling, likely through an imbalance introduced by non-peptidic moieties. Going forwards, new tools/technologies, as well as triple agonists and GIPR antagonists are likely to further illuminate the mechanisms of GLP1R and GIPR signalling in islets.

## Figures and Tables

**Table 1:** GLP1R expression patterns and levels within the different islet cell types

Cell type	*GLP1R/Glp1r* mRNA	GLP1R/Glp1r protein
Beta cell	**+++**	**+++**
Alpha cell	**-**	**-**
Delta cell	**++**	**-**

**Table 2:** GIPR expression patterns and levels within the different islet cell types

Cell type	*GIPR/Gipr* mRNA	GIPR/Gipr protein
Beta cell	**+++**	**++**
Alpha cell	****++**+**	**++**
Delta cell	**++**	?

## Highlights

- GLP1R is largely expressed in pancreatic beta cells and (possibly) delta cells.
- GIPR is expressed in beta cells, alpha cells and (possibly) delta cells.
- G_s_-coupled GLP1R and GIPR regulate insulin, glucagon and somatostatin release.
- Stabilized agonists targeting GLP1R are widely used in type 2 diabetes therapy.
- Dual agonists are imbalanced toward GIPR, which synergizes with GLP1R signaling.
